# Multivariate Data Analysis Methods and Their Application in Lipidomics: A Gentle Comment on Appropriateness and Reliability Criteria

**DOI:** 10.1111/jpi.70068

**Published:** 2025-07-21

**Authors:** Anna Migni, Desirée Bartolini, Giada Marcantonini, Roccaldo Sardella, Mario Rende, Alessia Tognoloni, Maria Rachele Ceccarini, Francesco Galli

**Affiliations:** ^1^ Department of Pharmaceutical Sciences University of Perugia Perugia Italy; ^2^ Department of Medicine and Surgery University of Perugia Perugia Italy

## Abstract

In response to Yoshiyasu Takefuji's critique regarding the use of Principal Component Analysis (PCA) and Partial Least Squares Discriminant Analysis (PLS‐DA) in the study “Melatonin Repairs the Lipidome of Human Hepatocytes Exposed to Cd and Free Fatty Acid‐Induced Lipotoxicity,” we provide a methodological clarification. PCA and PLS‐DA are well‐established, widely validated tools for exploratory analysis of high‐dimensional omics data, including lipidomics data. Although these methods are linear, they are appropriate for capturing systematic and directional variations in complex biological systems, particularly in controlled in vitro models like ours. Our analytical approach integrates PCA and PLS‐DA with rigorous statistical testing, data transformations, and biological validation, ensuring robustness and biological relevance of the findings. We reaffirm that these methods represent a standard, reliable practice in lipidomics, and the potential of nonlinear techniques does not diminish the appropriateness or utility of linear multivariate models when applied with scientific rigor.

In a recent letter to the Editor [1] Yoshiyasu Takefuji raised some issues about the methodology used to analyse the lipomics data in our article “Melatonin Repairs the Lipidome of Human Hepatocytes Exposed to Cd and Free Fatty Acid‐Induced Lipotoxicity” [2]. The critique specifically concerned an alleged misuse of Principal Component Analysis (PCA) and Partial Least Square Discriminant Analysis (PLS‐DA) methods to evaluate and interpret omics results.

In this commentary, we would like to address the concerns raised by Dr. Takefuji starting from the consideration that PCA and PLS‐DA are well‐established tools in the omics field that have extensively been validated for their application in data analysis and interpretation of metabolomics and lipidomics studies. These techniques are considered gold standards for pattern recognition, dimensionality reduction, and data visualization in high‐dimensional datasets. Their widespread use in hundreds of peer‐reviewed studies underscores their scientific robustness and interpretative value, particularly in exploratory data analysis (reviewed in [3, 4, 5, 6, 7, 8, 9, 10, 11, 12] and references therein).

The criticism that these methods are inappropriate due to their linear nature, misses a key nuance. Biological systems are neither strictly linear nor entirely nonlinear—they are complex, multivariate, and often exhibit semi‐linear behaviour. This complexity does not preclude the use of linear models for specific analytical purposes. On the contrary, in the context of cellular lipidomic data—even when derived from the same cell line under different treatments—systematic and directional metabolic changes can be effectively captured by linear multivariate models. PCA and PLS‐DA are therefore entirely appropriate as exploratory tools in such contexts [13, 14, 15, 16, 17].

Our study focused on a well‐defined in vitro model in which a single cell line (HepaRG) was exposed to specific treatments (e.g., melatonin, cadmium, fatty acids), each with clearly defined controls. In such controlled settings, lipidomic alterations tend to be systematic and directional, often reflecting changes in distinct metabolic pathways (e.g., FA, TG, CE, PE synthesis). These structured variations are well captured by multivariate linear techniques such as PCA and PLS‐DA, which enabled us to isolate treatment‐specific lipidomic changes.

Far from being simplistic, our analytical strategy was carefully designed to balance biological complexity with clarity and robustness in the interpretation of treatment‐induced changes. Although cellular metabolism is inherently nonlinear, relative differences between experimental groups under controlled conditions can be accurately modelled using linear approaches—particularly when supported by appropriate data preprocessing, including log2 transformation and median‐centering as shown in distribution plots that visually confirm the effectiveness of normalization and the suitability of our data set for linear multivariate analysis (Figure 1). This approach highlights lipids that vary most between study groups without distorting their original biological variability.

**Figure 1:**
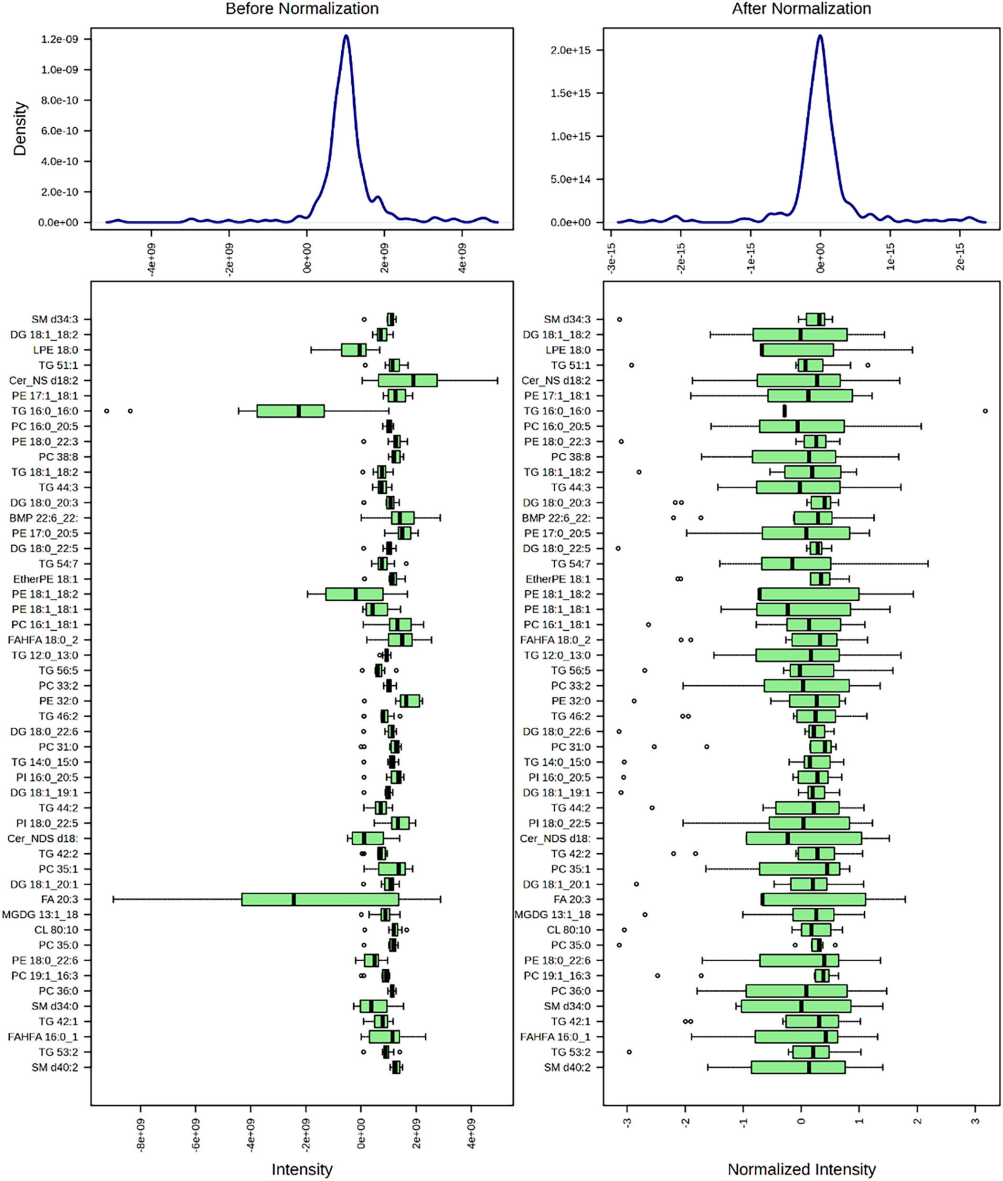
Lipidomics data distribution after transformation and scaling. Lipidomics data were normalized, then log2‐transformed and median‐centered.

Importantly, PCA and PLS‐DA were not the only strategies used in our data analysis. These served to preliminary explore and visualize the distribution of our cellular lipidome data in the different treatments. They were integrated into a broader and rigorous analytical pipeline, which included stringent data filtering (e.g., presence of the signal in ≥ 75% of samples with an intensity of at least 5000 counts), transformation and scaling, comprehensive univariate analyses (moderated *t*‐tests with FDR correction, *p* < 0.01, fold change > 1.5), multiple visualization strategies (volcano plots with data scattering and identification of differentially expressed annotations), and then biochemical and functional validation of omics results.

This multi‐level approach provided robust cross‐validation of experimental data ensuring both statistical and biological significance to their interpretation, and the fact that the results were consistent with the known effects of cadmium and melatonin on lipid metabolism of human hepatocytes ([2, 18] and references therein), further reinforced the relevance of our findings. All these steps adopted in our data analysis and interpretation strategy (summarized in Table 1) embody a standardized protocol adopted with minor variations in all the specialized omics labs and research groups active in the field of lipidomics. Specialized software developed to support lipidomics studies, are equipped with packages that follow this data analysis strategy [13] thus demonstrating its reliability and acceptance through the scientific community.

**Table 1:** PCA and PLS‐DA in omics data analysis and exploration: Methodological Considerations.

Steps/considerations	Description	References
1	PCA and PLS‐DA are standardized exploratory tools widely used in lipidomics and other omics disciplines.	[15, 16, 22]
2	Although they rely on assumptions of linearity, their main purpose is the visualization and synthesis of variance in high‐dimensional data, not statistical inference.	[15, 16, 22]
3	Data are log‐transformed and centgered to meet the analysis model assumptions. These transformations mitigate nonlinearity and scale differences, ensuring PCA and PLS‐DA are appropriate in the context of omics data analysis.	[22, 23]
4	Multivariate analysis is complemented by robust statistical testing (e.g., one‐way ANOVA or moderated *t*‐test with FDR).	[15, 16, 22, 24]
5	Conclusions on data analysis and interpretation are drawn from an integrated view of the results that should include biological and molecular validations of omics data.	[2, 24, 25]

In summary, we believe that our analytical approach is not misapplied, nor does it result in flawed or distorted interpretations as claimed in the critique. It is grounded in validated and robust methodologies, supported by comprehensive preprocessing, and applied in a biologically meaningful context. The use of PCA and PLS‐DA represents a well‐established and widely accepted practice in the omics field, particularly for exploratory analysis of high‐dimensional data in drug discovery and biomedical studies [19, 20, 21].

While we acknowledge the potential of nonlinear and machine learning techniques, we emphasize that this does not diminish the appropriateness and utility of linear multivariate methods in omics science, when applied rigorously and thoughtfully.

## Author Contributions

Anna Migni, Desirée Bartolini, and Francesco Galli conceived the commentary and drafted the initial manuscript. Anna Migni, Desirée Bartolini, Giada Marcantonini, Alessia Tognoloni, Maria Rachele Ceccarini, Francesco Galli, Roccaldo Sardella, and Mario Rende reviewed and revised the manuscript. All authors approved the final version of the manuscript as submitted.

## Conflicts of Interest

The authors declare no conflicts of interest.

## Data Availability

The data that support the findings of this study are available from the corresponding author upon reasonable request.
